# Applicability of TIVAP versus PICC in non-hematological malignancies patients: A meta-analysis and systematic review

**DOI:** 10.1371/journal.pone.0255473

**Published:** 2021-08-03

**Authors:** Baiying Liu, Zhiwei Wu, Changwei Lin, Liang Li, Xuechun Kuang

**Affiliations:** 1 Department of Gastrointestinal Surgery, The Third XiangYa Hospital of Central South University, Changsha, China; 2 Department of Geratic Surgery, Xiangya Hospital of Central South University, Changsha, Hunan, China; Ohio State University Wexner Medical Center Department of Surgery, UNITED STATES

## Abstract

**Background:**

Applicability of totally implantable venous access port (TIVAP) and peripherally inserted central venous catheter (PICC) in non-hematological malignancies patients remains controversial.

**Methods:**

A systematic studies search in the public databases PubMed, EMBASE, Wan Fang, CNKI (China National Knowledge Infrastructure), the Cochrane Library and Google Scholar (updated to May 1, 2020) was performed to identify eligible researches. All statistical tests in this meta-analysis were performed using Stata 12.0 software (Stata Corp, College Station, TX). A P value less than 0.05 was considered statistically significant.

**Results:**

Thirteen studies were included in this final meta-analysis. The pooled data showed that compared with PICC, TIVAP was associated with a higher first-puncture success rate (OR:2.028, 95%CI:1.25–3.289, P<0.05), a lower accidental removal rate (OR:0.447, 95%CI:0.225–0.889, P<0.05) and lower complication rates, including infection (OR:0.570, 95%CI: 0.383–0.850, P<0.05), occlusion (OR:0.172, 95%CI:0.092–0.324, P<0.05), malposition (OR:0.279, 95%CI:0.128–0.608, P<0.05), thrombosis (OR:0.191, 95%CI, 0.111–0.329, P<0.05), phlebitis (OR:0.102, 95%CI, 0.038–0.273, P<0.05), allergy (OR:0.155, 95%CI:0.035–0.696, P<0.05). However, no difference was found in catheter life span (P>0.05) and extravasation (P>0.05). Moreover, TIVAP is more expensive compared with PICC in six-month use (weighted mean difference:3.132, 95%CI:2.434–3.83, P<0.05), but is much similar in 12 months use (P>0.05).

**Conclusion:**

For the patients with non-hematological malignancies, TIVAP was superior to PICC in the data related to placement and the incidence of complications. Meanwhile, TIVAP is more expensive compared with PICC in six-month use, but it is much similar in twelve-month use.

## Introduction

With rapid population growth and aging societies worldwide, cancer has become the leading cause of death [1]. The process of treating oncology patients, however, is complicated [2]. Chemotherapy is one of the major treatments used to prolong the life span and to improve the quality of life of patients with cancer. Many chemotherapies, such as cytotoxic drugs infused through intravenous access, can lead to damage of the peripheral blood vessels. As such, establishing and maintaining good venous access is essential [3].

Currently, venous access includes peripheral venous and central venous routes. However, traditional peripheral venous access requires repeated venipuncture, which can aggravate patient pain and anxiety [4,5]. Central venous catheters (CVCs) represent a major advance for oncology patients, enabling the effective delivery of chemotherapy and blood products, particularly for long-term infusions or in situations involving difficult venous access [6]. Totally implantable venous access port (TIVAP) and peripherally inserted central venous catheters (PICCs) have been used worldwide as two integral components of state-of-the-art methods of CVCs [7–9]. However, they have different placement requirements and clinical effects.

TIVAP, which was introduced in the 1980s [10], is implanted using either an open surgical procedure or by an imaging-guided radiological intervention [11,12], without the requirement for external catheter lines [13]. It affords adequate patient comfort with minimal restriction to normal activities and an acceptable cosmetic result [14,15]. PICCs are usually inserted through a superficial vein in the upper extremity [8,14]. They are inserted and removed either by interventional radiologists or other advanced practice providers like vascular access nurses under ultrasound or fluoroscopic guidance and afford quicker and more accessible management [16–18]. Many studies have analyzed the advantages and disadvantages of the two methods. However, current published studies have not been well-balanced, and the majority of included patients were diagnosed with hematological malignancies. Chemotherapy for these patients is more likely to cause myelosuppression compared to those with non-hematological malignancies [19]. A previous study indicated that hematological malignancies are an independent risk factor for complications in patients with an external access device [6]. There is no clear or consistent evidence as to which type is safer or preferable in those with non-hematological malignancies. As such, the evidence supporting the choice of one design over the other remains controversial. Accordingly, we conducted a meta-analysis of all available—and ultimately, eligible—studies to investigate and evaluate the data related to placement, complications, and PICC line versus TIVAP devices in patients with non-hematological malignancies.

## Methods

### Search strategies

The studies related to applications of PICC versus TIVAP in non-hematological malignancies with chemotherapy were searched in the databases of PubMed, EMBASE, Wan Fang, CNKI, the Cochrane Library and Google Scholar using the keywords of ("totally implantable vascular access device " OR "PORT" OR "Port-A-Cath" OR "PAC" OR "venous access port" OR "VAP" OR "totally implantable venous access port" OR "TIVAP" OR "TIVAD" OR "venous port access" OR "VPA" OR "central venous access device" OR"CVAD" AND "Catheterization, Peripheral" [Mesh]). All included articles were published before May 1 2020. An additional relevant search was performed by manually searching the references of eligible studies or relevant reviews. The articles were selected by two reviewers independently. Any inconsistent opinion was solved by discussion and re-evaluation until a consensus was built.

### Selection criteria

The publication inclusion criteria: 1) prospective or retrospective clinical research; 2) adult patients diagnosed with non-hematology cancer; 3) the research must contain TIVAP group and PICC group; 4) one or more observation indicators are required, including first-puncture success rate, complications like occlusion, infection, malposition, catheter-related thrombosis, phlebitis, and accidental removal rate and cost analysis; 5) the language of the included articles are English or Chinese.

The publication exclusion criteria: 1) the research objects included in the experiments contained hematological cancer; 2) review and case report were excluded; 3) failed to compare two methods; 4) repeated data from the same population.

### Quality assessment

The quality of each study was assessed using The Newcastle Ottawa Assessment Scale (NOQAS) by two independent reviewers [20]. A maximum of nine-point scales for quality of selection, comparability, and outcome of study participants was allocated to assess the quality of observational studies. Studies that scored 6 of the 9 points were considered to be included. NOQAS scores of each study in this meta-analysis ranged from 6 to 9.

### Data extraction

All data were extracted by two independent reviewers. Discrepancies were resolved by discussion and re-evaluation. We extracted the basic study information (name of the first author, year of publication, region or country where the study was conducted and study population), participant characteristics (gender), details of observation group and control group, including the type of cancer; puncture and remove catheters; type of complications and costs.

### Statistical analysis

Odds ratio (OR) was used to calculate the binary variable and weighted mean difference to analyze continuous data. For studies that did not include the point estimates, we used available data and applied the method reported by Tierney et al. to determine the OR and its 95% confidence interval (CI) [21]. When OR>1, it means that TIVAP has a higher risk than PICC. When OR<1, it means that TIVAP has a lower risk than PICC. When OR = 1 or 95% CI across 1, it means that there is no significant difference between TIVAP and PICC. Weighted mean difference was used to validate continuous variables such as catheter life span and cost. Heterogeneity across studies was checked by a chi-square based on Q test and the I^2^ test. I^2^ values<25% is an indicator of mild heterogeneity, I^2^ values between 25% and 50% correspond to moderate heterogeneity, and I^2^ values>50% correspond to large heterogeneity. For a Q statistic P-value≥0.05 or I^2^<50%, we used a fixed-effects model to calculate the pooled estimates; otherwise, a more conservative random-effects model was used. Sensitivity analysis was performed to test the reliability of the overall pooled results. All statistical tests in this meta-analysis were performed using Stata 12.0 software (Stata Corp, College Station, TX). A P value less than 0.05 was considered statistically significant.

### Statement

Our work has been reported in line with PRISMA (Preferred Reporting Items for Systematic Reviews and Meta-Analyses) and AMSTAR (Assessing the methodological quality of systematic reviews) Guidelines.

## Results

### Search results and study characteristics

**Fig 1:**
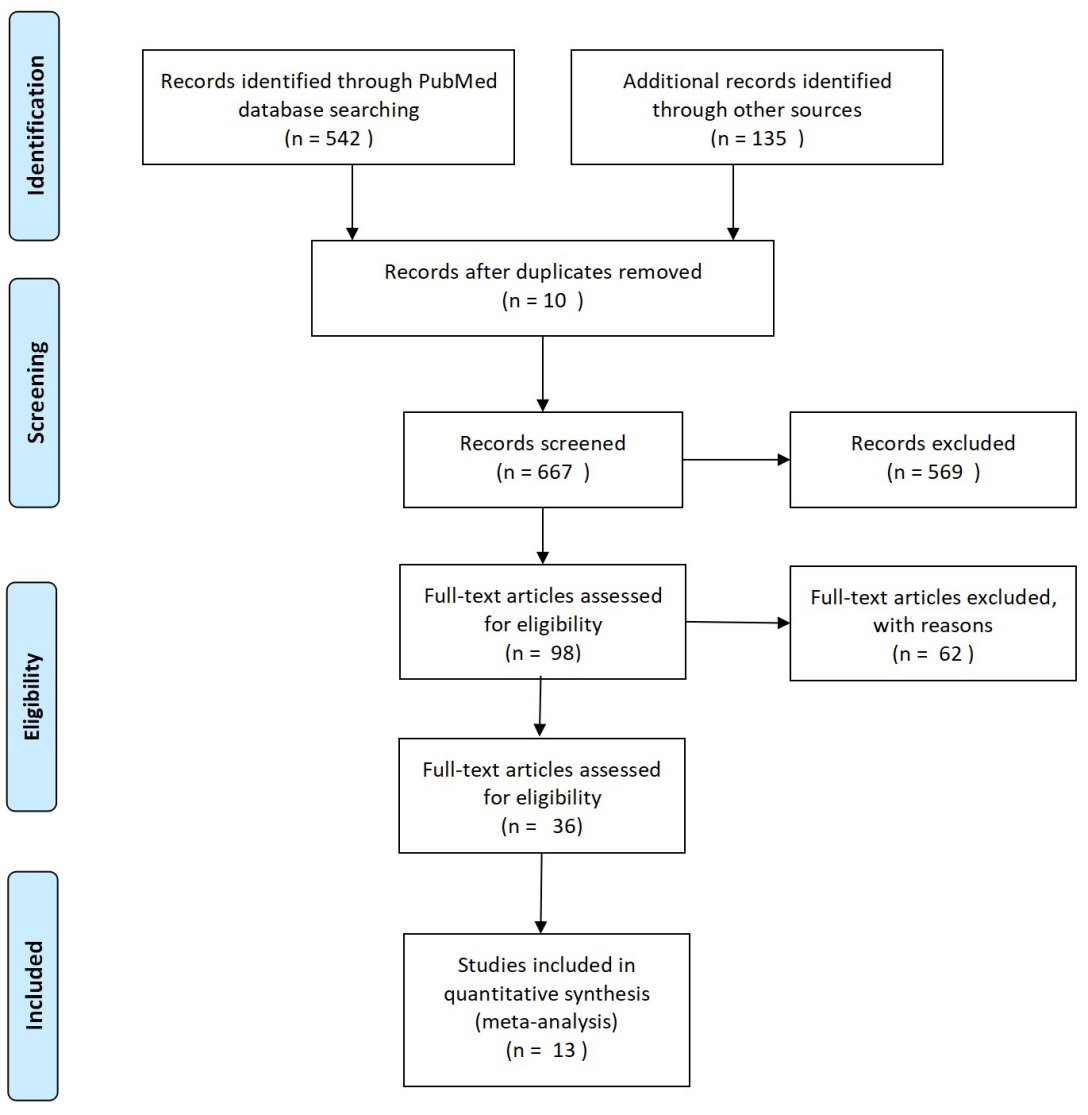
Prisma®flow diagram of the searching processes and results.

The initial literature search retrieved 669 potentially eligible studies. After screening titles, however, 536 records were excluded: 463 were non-cancer related, 50 were reviews, letters, or case reports, and 10 were duplicates. Among the remaining 133 articles, 92 were excluded after reading the abstract, including 41 that failed to address PICC or TIVAP and 28 that investigated patients with hematological cancers. After full-text review, 28 additional articles were excluded due to lack of useful outcome(s) and one for data repeated from the same population. Ultimately, 13 cohort studies involving 3,239 patients [2,3,6,22–31], based on the inclusion criteria and quality assessment, were included. Details of the selection process are illustrated in Fig 1. Among these 13 studies, six were from China, five from Europe, one each was from Canada and Australia. Table 1 summarized the detailed characteristics of these studies.

**Table 1:** Characteristics of 13 studies included in the meta-analysis.

Author/Year	Country	Journal	Cases(n)	Gender(M/F)	NOS score
Knut et al. [22]/2019	Sweden	Br J Anaesth	399	174/225	8
Wang et al. [26]/2016	China	Med Philos B	110	0/110	7
Verboom et al. [3]/2017	Netherlands	Clin Sarcoma Res	112	NA	7
Wang et al. [30]/2019	China	Chinese Journal of Hospital Statistics	240	0/240	7
Hou et al. [31]/2017	China	Chinese Journal of Woman and Child Health Research	725	0/725	8
Lefebvre et al. [27]/2016	Germany	Support Care Cancer	448	0/448	9
Clemons et al. [23]/2020	Canada	Supportive Care in Cancer	41	0/41	8
Fang et al. [25]/2017	China	Patient Prefer Adherence	105	45/60	8
Lu et al. [28]/2017	China	Chin Remedies Clin	550	0/550	7
Liu et al. [29]/2017	China	Chin J Prac Nurs	298	0/298	7
Patel et al. [6]/2013	Australia	Supportive Care in Cancer	70	36/34	8
Martella et al. [24]/2015	Italian	Anticancer Drugs	102	4/98	8
Coady et al. [2]/2015	United Kindoms	The journal of vascular access	39	15/24	7

NA: Not available.

### Quantitative synthesis

To evaluate the applicability of TIVAP and PICC in non-hematological cancer patients, relevant data were investigated in the present meta-analysis. Data regarding first-puncture success rate, accidental removal rate, complications, such as occlusion, infection, malposition, thrombosis, extravasation, phlebitis and allergy, were extracted from the included studies for the calculation of pooled odds ratios (ORs). Catheter life span and cost(s) were extracted to calculate pooled mean differences.

### Data related to placement

#### First-puncture success rate

**Fig 2:**
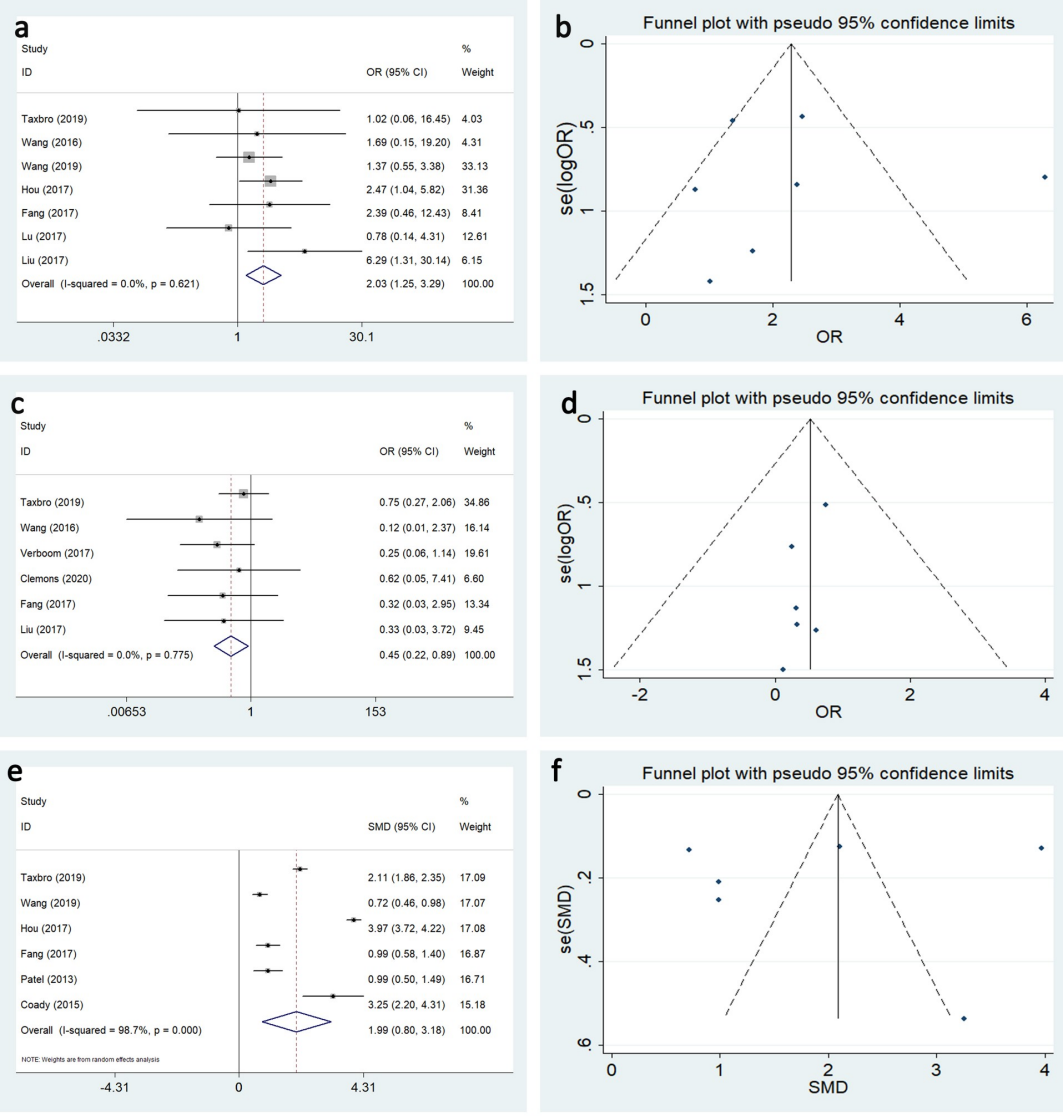
Forest plots and Funnel plots for publication bias test for the comparing data related to placement between TIVAP and PICC. (a and b) the first-puncture success rate, (c and d) the accidental removal rate, (e and f) catheter life span.

Seven studies reported the effect of TIVAP and PICC on the first-puncture success rate. There was no significant heterogeneity among the studies (I^2^ = 0.0%, P = 0.621), and the results revealed that the first-puncture success rate was significantly higher than that for PICC (OR:2.028, 95%CI:1.25–3.289, P = 0.004) (Fig 2A and 2B).

#### Accidental removal rate

Six studies reported the effects of TIVAP and PICC on the accidental removal rate. No statistical heterogeneity was observed among the studies (I^2^ = 0.0%, P = 0.775). Analysis revealed that the accidental removal rate was significantly lower for TIVAP compared with PICC (OR:0.447, 95%CI:0.225–0.889, P = 0.022) (Fig 2C and 2D).

#### Catheter life span

Results from six studies were pooled to analyze the effect of TIVAP and PICC on catheter life span. Apparent heterogeneity was clear (I^2^ = 98.7%); as such, a random effect model was used with the results (OR:1.988, 95%CI:0.796–3.180, P = 0.001) (Fig 2E and 2F). Although the catheter life span for TIVAP was longer than that for PICC, there were no statistical differences between the two methods.

#### Complications

**Fig 3:**
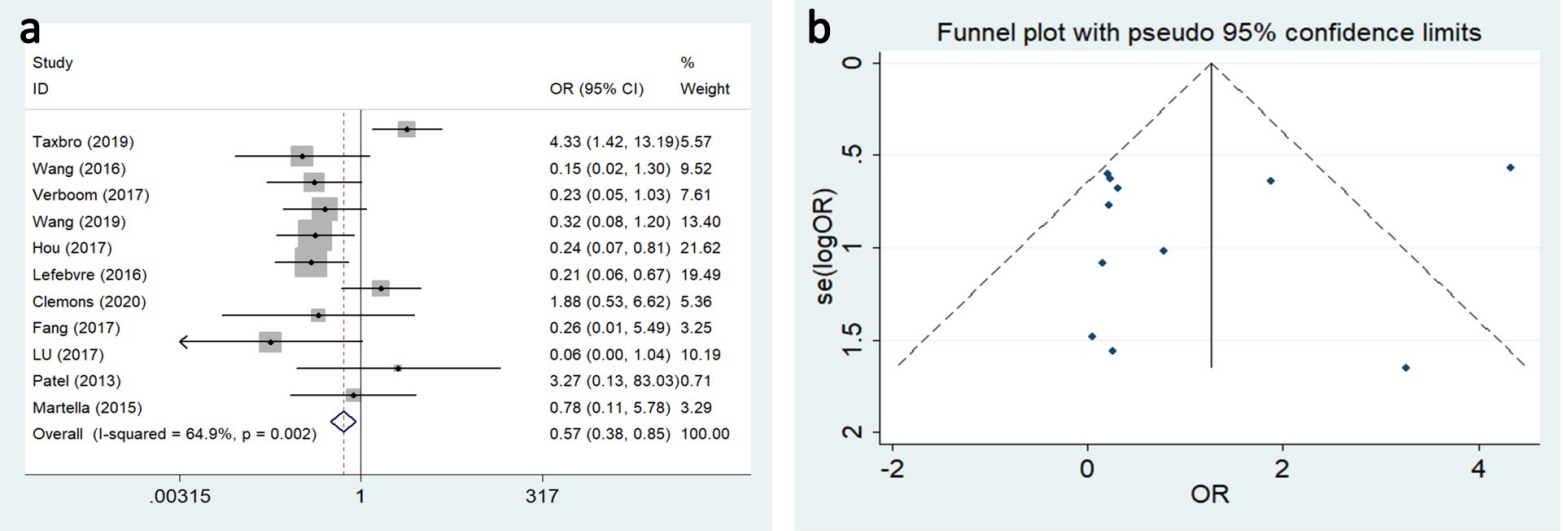
Forest plots and funnel plot for publication bias test for the comparing the occlusion between TIVAP and PICC.

Catheter-related complication rates ranged from 3.5% to 19% in the TIVAP group, and from 12% to 37% in the PICC cohorts, and mainly included infection, occlusion, malposition, thrombosis, extravasation, phlebitis and allergy. The most common complication was infection. Significant results with moderate between-study heterogeneity indicated that TIVAP was associated with a lower infection rate compared with PICC (OR: 0.570, 95%CI:0.383–0.850, P = 0.006, I^2^ = 64.9%) (Fig 3).

**Fig 4:**
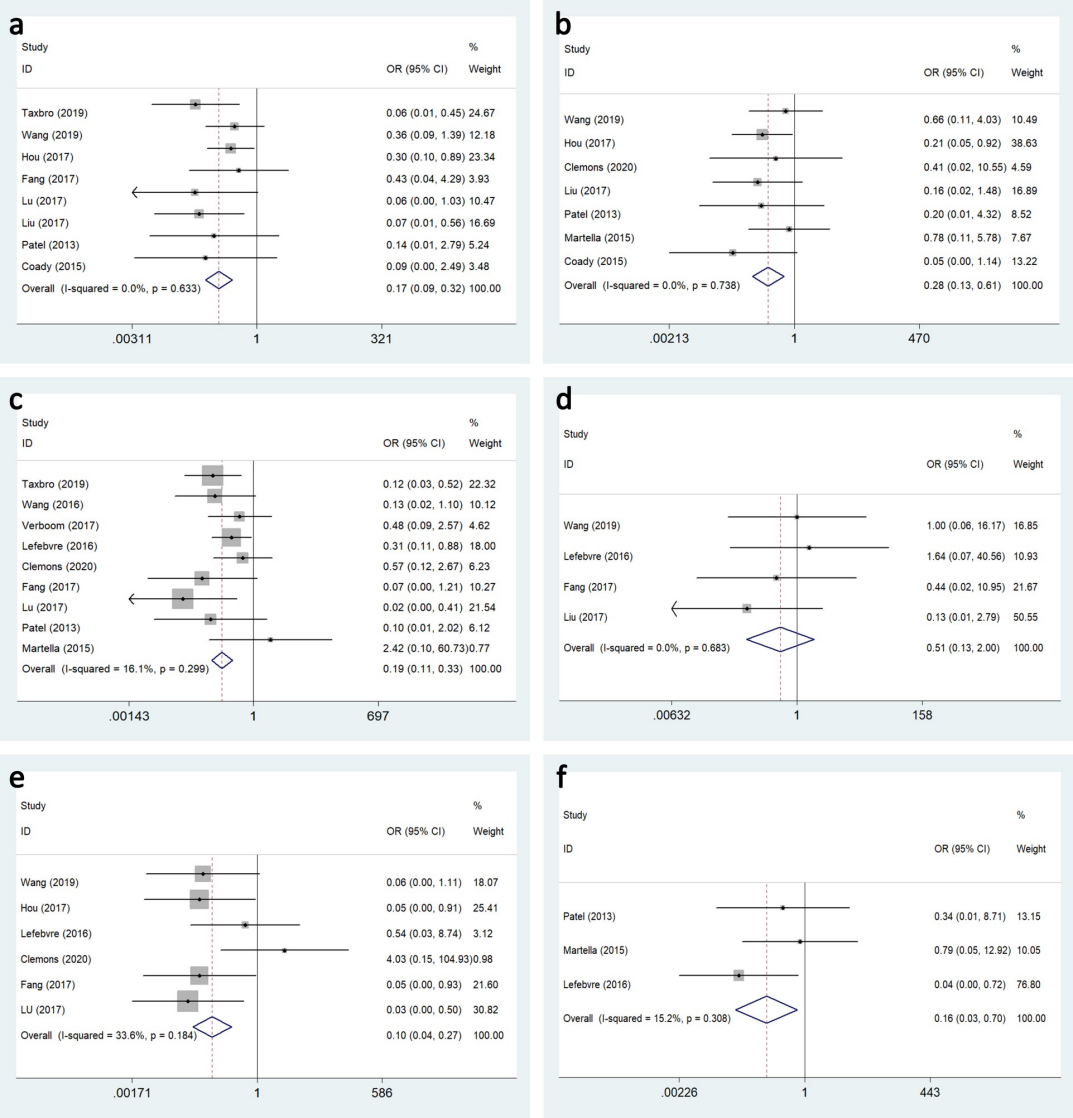
Forest plot for the relationship of TIVAP and PICC with the risk of follow complications. (a)occlusion, (b) malposition, (c) catheter-related thrombosis, (d) extravasation, (e)phlebitis, (f) allergy.

Occlusion in the TIVAP group was significantly lower than in the PICC group (OR:0.172, 95%CI:0.092–0.324) (Fig 4A), and malposition (OR:0.279, 95%CI:0.128–0.608) (Fig 4B), thrombosis (OR:0.191, 95%CI:0.111–0.329) (Fig 4C), phlebitis (OR:0.102, 95%CI:0.038–0.273) (Fig 4E), and allergy (OR:0.155, 95%CI:0.035–0.696) (Fig 4F). However, there was no significant difference between the TIVAP and PICC groups with regard to extravasation (OR:0.510, 95%CI:0.130–1.997) (Fig 4D).

**Fig 5:**
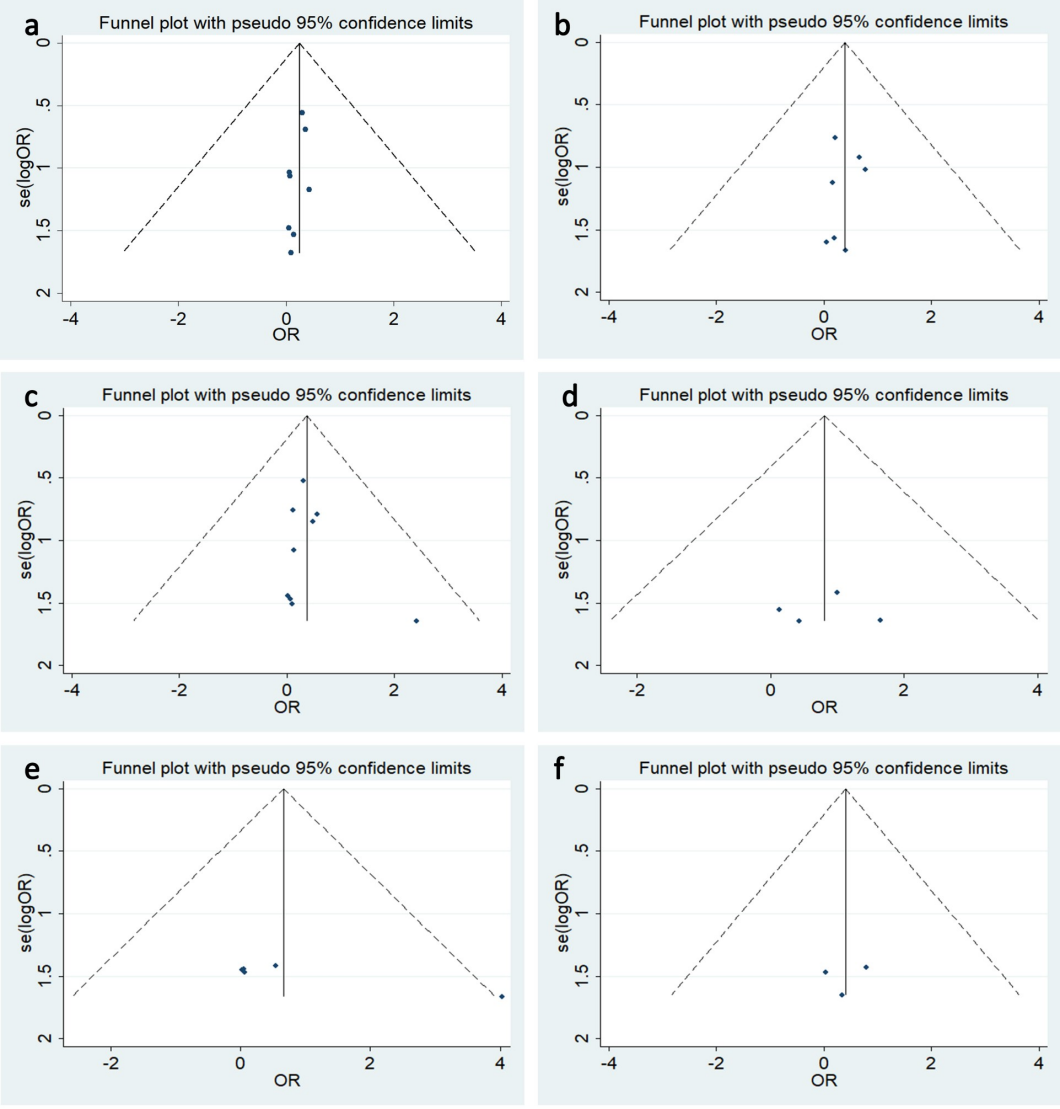
Funnel plot for publication bias test for the relationship of TIVAP and PICC with the risk of follow complications. (a)occlusion, (b) malposition, (c) catheter-related thrombosis, (d) extravasation, (e)phlebitis, (f) allergy.

Publication bias in the selected studies was assessed using funnel plot and Begg’s test. There was moderate statistical between-study heterogeneity for phlebitis (I^**2**^ = 33.6%) and mild heterogeneity for thrombosis (I^**2**^ = 16.1%) and allergy (I^**2**^ = 15.2%). No evidence of publication bias was found among the other complications (Fig 5).

#### Costs

**Fig 6:**
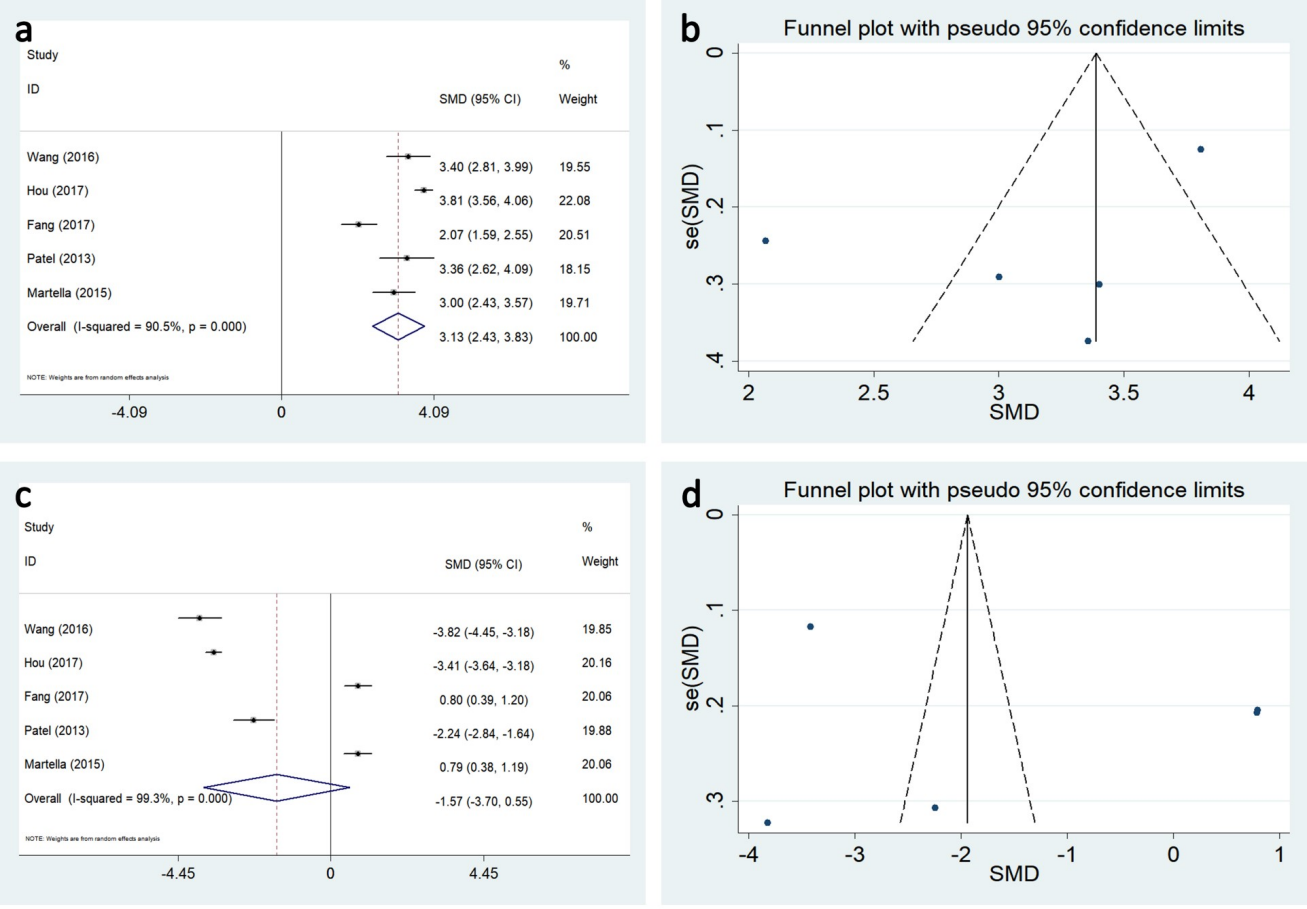
Forest plots and funnel plots for publication bias test for the comparing data related to cost between TIVAP and PICC. (a and b) the catheters maintain less than 6 months, (c and d) the catheter duration more than 12 months.

Pooled meta-analysis was conducted using five studies. When the catheter was maintained for < 6 months, the cost was significantly higher in the TIVAP group than in the PICC group (weighted mean difference:3.132, 95%CI 2.434–3.83, P < 0.05) (Fig 6A and 6B). However, when the catheter indwell duration was > 12 months, although the cost of TIVAP is supposed to be lower than PICC with prolonged use, the cost between the two methods was similar (weighted mean difference:-1.574, 95%CI: -3.699–0.551, P > 0.05) ((Fig 6C and 6D).

## Discussion

TIVAP and PICCs are two integral components methods of CVCs, widely used in oncology patients management. Many studies have been conduct to compare the application value between TIVAP and PICC in oncology patients management. Previous similar meta-analyses have often included patients with hematological malignancies [32], which patient population usually exhibits heterogeneity in severe myelosuppression. Therefore, the meta-analysis of patients with hematological malignancies choosing TIVAP or PICC as their better central venous access cannot provide a practical guide for non-hematological cancer patients. Patients with non-hematological cancer(s) still, however, must choose one type of specific vascular access for oncology management. Whether PICC or TIVAP is a better choice for management in non-hematological cancer patients remains controversial.

Accordingly, we conducted a meta-analysis and found that for data related to placement, TIVAP was associated with a higher first-puncture success rate and lower accidental removal rate compared with PICC, and there was no significant difference in catheter life span between the two methods. Second, TIVAP was associated with lower complication rates than PICC, including infection, occlusion, malposition, catheter-related thrombosis, phlebitis, and accidental removal rate. However, no significant difference was found in extravasation. Third, TIVAP was more expensive compared with PICC in six-month use, but was essentially similar over a 12-month period and may account for lower costs than PICC when used for > 12 months.

Nowadays, ultrasound and X-ray greatly facilitated the line construction. Standardized training for line placement also improved the puncture success rate. However, whether TIVAP or PICC has a higher first-puncture rate remains controversial. Many studies have reported that the first-puncture success rate for TIVAP is higher than that for PICC; however, Lu et al. reported a higher first-puncture success rate for PICC with no significant difference between TIVAP and PICC [28]. We conducted this meta-analysis and found that TIVAP was associated with a higher first-puncture success rate. Meanwhile, we found that the removal rate in the TIVAP group was lower than in the PICC group, which is consistent with results from most studies.

TIVAP could theoretically remain in vivo for 19–38 years. It demands simple maintenance once per month and functions for many years. Although catheter life span has been reported to be longer in TIVAP than in PICC in most studies, we found no significant difference in catheter life span between the two methods in the present meta-analysis. Most non-hematology cancer patients choose to have their catheters removed after chemotherapy, which may contribute to this result. Moreover, there are fewer complications associated with TIVAP maintenance.

Seven complications were addressed in our study, including infection, occlusion, malposition, catheter-related thrombosis, extravasation, phlebitis and allergy. Taxbro et al. reported a higher risk for infection in the TIVAP group [22]. However, Verboom et al. suggested that the infection rate for TIVAP is lower than that for PICC [3]. Our meta-analysis revealed that the incidence of TIVAP-related infection was lower than that for PICC. The puncture point for PICC is the upper arm and catheter joints are vulnerable to infection. However, TIVAP has no external exposure because its catheter is totally implanted under the skin; therefore, the infection rate is comparable with that of PICC. We also found that the incidence of occlusion, malposition, catheter-related thrombosis, phlebitis and allergy were significantly lower in the TIVAP group than in the PICC group. Thrombosis and line occlusion are severe complications in cancer patients with venous catheters. According to related studies, catheter occlusion is more likely to occur in patients with PICC lines [33]. A possible reason is that PICCs are placed in small vessels, resulting in a large percentage of vessel lumen occupied by the catheter, which may cause stasis of flow and thrombosis. The increased mobility of PICCs also stimulates blood vessels and causes endothelial injury and leads to phlebitis. Lefebvre et al. reported two types of malposition: catheter dislodgement and migration of the tip [27]. Moreover, they also documented more PICC-related malposition in their study. A possible reason is that PICCs need to be inserted from the median vein cubits to the superior vena cava. The branches of the superior vena cava are complicated; as such, it is difficult for the PICC tip to be installed at an ideal position without a guide conductor. Glauser et al. introduced a fluoroscopically guided technique for PICC that reduced tip malposition rate at insertion [34]. However, TIVAP is usually inserted in the operating room using Doppler ultrasound guidance and is securely fixed under the skin after insertion. Therefore, it is not easy to be moved or dislodged by the activities of daily life. The visualized operation assisted by doppler ultrasound can minimize the difficulty of TIVAP placement and thus, reduce relevant complications [35]. The most common reason for allergy is dressing. PICC restricts the choice of dressing and the puncture site, which is always in the arm skin contact with the dressing. It is more frictive in daily life than TIVAP, for which the puncture site is usually located in the chest. Therefore, PICC caused more allergic problems reported in those studies. Our meta-analysis revealed no significant difference in extravasation between the TIVAP and PICC groups, consistent with a previous study. Moreover, we noticed some specific complications when inserting TIVAP, including pneumothorax, arterial puncture, and other serious issues. These complications were mainly associated with physician proficiency in the operation/procedure; therefore, technical training and accumulation of relevant experience are essential for successful TIVAP implantation.

Regarding costs associated with the two methods, the results of our analysis suggest that TIVAP is more expensive than PICC in 6-month use, but is highly similar over a 12-month period, and may actually account for lower costs than PICCs used over 12 months. Most non-hematological cancer patients end their chemotherapy in 9 months [24], and PICC is more efficient in mitigating major complications because it can be removed at the bedside compared with TIVAP, which requires a one-day surgical procedure. When the regimen is over 1 year, TIVAP is easier to maintain with regard to dressing changes (every four weeks) compared with PICC (every week). As such, it dramatically reduces transport expenses and other relative costs to patients visiting the hospital for necessary maintenance.

## Limitation

Publication limitation could have been present due to the inclusion of English and Chinese published studies only. So geography bias may exist. Meanwhile, PICC is used for a short duration, while TIVAP is used for a longer period. The AEs(adverse events) among the two methods in terms of both short period (for example, 3 weeks) and long period (for example, 3 months) are lack in previous studies, further studies need to compare this in the future. Additionally, the cost may be diverse in different regions, as many other factors can influence the cost. Further, the satisfaction and comfort of patients with TIVAP or PICC were not evaluated in our meta-analysis for the different criteria difficult to conduct and lack of sufficient data. Moreover, heterogeneity was also observed in this meta-analysis, resulting from different characteristics of the included patients and inconsistent standards of some evaluation parameters.

## Conclusion

According to the results of our meta-analysis, for the patients with non-hematological malignancies, TIVAP was superior to PICC in the data related to placement and the incidence of complications. TIVAP is more expensive compared with PICC in six-month use, but is much similar in 12 months.

## Supporting information

S1 Checklist PRISMA 2009 checklist. (DOC)

S1 Fig Flow diagram of the searching processes and results in PUBMED. (TIF)

S1 Table PUBMED search strategies and results. (DOC)

S1 File (DOC)

## References

[1] Bray F, Ferlay J, Soerjomataram I, Siegel RL, Torre LA, Jemal A. Global cancer statistics 2018: GLOBOCAN estimates of incidence and mortality worldwide for 36 cancers in 185 countries. CA Cancer J Clin. 2018;68(6):394–424. doi: 10.3322/caac.21492 30207593

[2] Coady K, Ali M, Sidloff D, Kenningham RR, Ahmed S. A comparison of infections and complications in central venous catheters in adults with solid tumours. The journal of vascular access. 2015;16(1):38–41. doi: 10.5301/jva.5000300 25198809

[3] Verboom MC, Ouwerkerk J, Steeghs N, Lutjeboer J, Martijn Kerst J, van der Graaf WTA, et al. Central venous access related adverse events after trabectedin infusions in soft tissue sarcoma patients; experience and management in a nationwide multi-center study. Clin Sarcoma Res. 2017;7:2–2. doi: 10.1186/s13569-017-0066-6 28163887 PMC5282803

[4] Silvestri V, Nerini L, Missio G, Masini M, Faggi S, Gori A, et al. Levels of anxiety and pain during chemotherapy with peripheral versus central vascular access: an experimental evaluation. The journal of vascular access. 2004;5(4):147–153. doi: 10.1177/112972980400500403 16596558

[5] Masoorli S. Nerve injuries related to vascular access insertion and assessment. J Infus Nurs. 2007;30(6):346–350. doi: 10.1097/01.NAN.0000300310.18648.b2 18025982

[6] Patel GS, Jain K, Kumar R, Strickland AH, Pellegrini L, Slavotinek J, et al. Comparison of peripherally inserted central venous catheters (PICC) versus subcutaneously implanted port-chamber catheters by complication and cost for patients receiving chemotherapy for non-haematological malignancies. Supportive care in cancer. 2014;22(1):121–128. doi: 10.1007/s00520-013-1941-1 24005884

[7] Smith JR, Friedell ML, Cheatham ML, Martin SP, Cohen MJ, Horowitz JD. Peripherally inserted central catheters revisited. Am J Surg. 1998;176(2):208–211. doi: 10.1016/s0002-9610(98)00121-4 9737634

[8] Gebauer B, Teichgräber UKM, Podrabsky P, Beck A, Wagner HJ. Ultrasound- and fluoroscopy-guided implantation of peripherally inserted central venous catheters (PICCs). Rofo. 2004;176(3):386–391. doi: 10.1055/s-2004-812737 15026952

[9] Niederhuber JE, Ensminger W, Gyves JW, Liepman M, Doan K, Cozzi E. Totally implanted venous and arterial access system to replace external catheters in cancer treatment. Surgery. 1982;92(4):706–712. 7123491

[10] Rotzinger R, Gebauer B, Schnapauff D, Streitparth F, Wieners G, Grieser C, et al. Placement of central venous port catheters and peripherally inserted central catheters in the routine clinical setting of a radiology department. analysis of costs and intervention duration learning curve. Acta Radiol. 2017;58(12):1468–1475. doi: 10.1177/0284185117695664 28406048

[11] Kock HJ, Pietsch M, Krause U, Wilke H, Eigler FW. Implantable vascular access systems: experience in 1500 patients with totally implanted central venous port systems. World J Surg. 1998;22(1):12–16. doi: 10.1007/s002689900342 9465755

[12] Adamus R, Beyer-Enke S, Otte P, Loose R. [Ultrasound-guided puncture of the subclavian vein to implant central venous ports]. Rofo. 2002;174(11):1450–1453. doi: 10.1055/s-2002-35352 12424674

[13] Gonda SJ, Li R. Principles of subcutaneous port placement. Tech Vasc Interv Radiol. 2011;14(4):198–203. doi: 10.1053/j.tvir.2011.05.007 22099011

[14] Teichgräber UK, Gebauer B, Benter T, Wagner J. [Long-term central venous lines and their complications]. Rofo. 2004;176(7):944–952. doi: 10.1055/s-2004-813258 15237335

[15] Bow EJ, Kilpatrick MG, Clinch JJ. Totally implantable venous access ports systems for patients receiving chemotherapy for solid tissue malignancies: A randomized controlled clinical trial examining the safety, efficacy, costs, and impact on quality of life. J Clin Oncol. 1999;17(4):1267. doi: 10.1200/JCO.1999.17.4.1267 10561188

[16] Gebauer B, Teichgräber UK, Podrabsky P, Beck A, Wagner HJ. [Ultrasound- and fluoroscopy-guided implantation of peripherally inserted central venous catheters (PICCs)]. Rofo. 2004;176(3):386–391. doi: 10.1055/s-2004-812737 15026952

[17] Pullyblank AM, Carey PD, Pearce SZ, Tanner AG, Guillou PJ, Monson JR. Comparison between peripherally implanted ports and externally sited catheters for long-term venous access. Ann R Coll Surg Engl. 1994;76(1):33–38. 8117017 PMC2502183

[18] Lindquester WS, Dhangana R, Warhadpande S, Amesur NB. Effects of the MAGIC Guidelines on PICC Placement Volume: Advanced Practice Provider and Physician Trends Among Medicare Beneficiaries From 2010 to 2018. AJR Am J Roentgenol. 2021;(24):1–5. doi: 10.2214/AJR.20.23704 32845711

[19] Hsieh C-C, Weng H-H, Huang W-S, Wang W-K, Kao C-L, Lu M-S, et al. Analysis of risk factors for central venous port failure in cancer patients. World J Gastroenterol. 2009;15(37):4709–4714. doi: 10.3748/wjg.15.4709 19787834 PMC2754519

[20] Wang Y, Mo Y, Yang X, Zhou R, Wu Z, He Y, et al. Long non-coding RNA AFAP1-AS1 is a novel biomarker in various cancers: a systematic review and meta-analysis based on the literature and GEO datasets. Oncotarget. 2017;8(60):102346–102360. doi: 10.18632/oncotarget.21830 29254250 PMC5731960

[21] Tierney JF, Stewart LA, Ghersi D, Burdett S, Sydes MR. Practical methods for incorporating summary time-to-event data into meta-analysis. Trials. 2007;8:16–16. doi: 10.1186/1745-6215-8-16 17555582 PMC1920534

[22] Taxbro K, Hammarskjold F, Thelin B, Lewin F, Hagman H, Hanberger H, et al. Clinical impact of peripherally inserted central catheters vs implanted port catheters in patients with cancer: an open-label, randomised, two-centre trial. Br J Anaesth. 2019;122(6):734–741. doi: 10.1016/j.bja.2019.01.038 31005243

[23] Clemons M, Stober C, Kehoe A, Bedard D, MacDonald F, Brunet M-C, et al. A randomized trial comparing vascular access strategies for patients receiving chemotherapy with trastuzumab for early-stage breast cancer. Support Care Cancer. 2020;Oct;28(10):4891–4899. doi: 10.1007/s00520-020-05326-y 32002617

[24] Martella F, Salutari V, Marchetti C, Pisano C, Di Napoli M, Pietta F, et al. A retrospective analysis of trabectedin infusion by peripherally inserted central venous catheters: a multicentric Italian experience. Anticancer Drugs. 2015;26(9):990–994. doi: 10.1097/CAD.0000000000000275 26241804

[25] Fang S, Yang J, Song L, Jiang Y, Liu Y. Comparison of three types of central venous catheters in patients with malignant tumor receiving chemotherapy. Patient Prefer Adherence. 2017;11:1197–1204. doi: 10.2147/PPA.S142556 28744109 PMC5513891

[26] Wang N, Dong Y, Zhang B, Gao YJ, Fu H. Comparison ofthe application of IVPA and PICC in breast cancer patients. Med Philos B. 2016;37(7):36–38. doi: 10.12014/j.issn.1002-0772.2016.07b.09

[27] Lefebvre L, Noyon E, Georgescu D, Proust V, Alexandru C, Leheurteur M, et al. Port catheter versus peripherally inserted central catheter for postoperative chemotherapy in early breast cancer: a retrospective analysis of 448 patients. Supportive care in cancer. 2016, 24(3):1397–1403. doi: 10.1007/s00520-015-2901-8 26342484

[28] Lu XT, Gao RF, Zhang YF. Clinical use of ultrasound-guided implantable venous access port versus PICC in chemotherapy of breast cancer. Chin Remedies Clin. 2017;17(1):13–16. doi: 10.11655/zgywylc2017.01.005

[29] Liu Y. Comparison of implanted vascular access ports and PICC in breast cancer patients. Chin JPracNurs. 2017;10(18):1413–1416. doi: 10.3760/cma.j.issn.1672-7088.2017.18.015

[30] Wang YL, Ma ZS, Lin J, Wu LL. Study on effect comparison between implantable venous access port and peripherally inserted central catheter of chemotherapy patients of breast cancer. Chinese Journal of Hospital Statistics. 2019;26(01):59–61. doi: 10.3969/j.issn.1006-5253.2019.01.017

[31] Hou NR, Chen J, Zhang HX, Yao WX. Application study of implantable venous port access and peripherally inserted central catheter on cervical cancer patients undergoing chemotherapy. Chinese Journal of Woman and Child Health Research. 2017; 28(09):1118–1121. doi: 10.3969/j.issn.1673-5293.2017.09.026

[32] Pu YL, Li ZS, Zhi XX, Shi YA, Meng AF, Cheng F, et al. Complications and Costs of Peripherally Inserted Central Venous Catheters Compared With Implantable Port Catheters for Cancer Patients: A Meta-analysis. Cancer Nurs. 2020;43(6):455–467. doi: 10.1097/NCC.0000000000000742 31464692

[33] Fallouh N, McGuirk HM, Flanders SA, Chopra V. Peripherally Inserted Central Catheter-associated Deep Vein Thrombosis: A Narrative Review. Am J Med. 2015; 128(7):722–738. doi: 10.1016/j.amjmed.2015.01.027 25697969

[34] Glauser F, Breault S, Rigamonti F, Sotiriadis C, Jouannic AM, Qanadli SD. Tip malposition of peripherally inserted central catheters: a prospective randomized controlled trial to compare bedside insertion to fluoroscopically guided placement. Eur Radiol. 2017;27(7):2843–2849. doi: 10.1007/s00330-016-4666-y 27957644

[35] Cajozzo M, Palumbo VD, Mannino V, Geraci G, Lo Monte AI, Caronia FP, et al. Ultrasound-guided port-a-cath positioning with the new one-shoot technique: thoracic complications. Clin Ter. 2018;169(6):e277–e280. doi: 10.7417/CT.2018.2093 30554248

