# Supplementary figures and images for: Applicability of TIVAP versus PICC in non-hematological malignancies patients: A meta-analysis and systematic review
Source: PLoS One. 2021 Aug 3;16(8):e0255473. doi: 10.1371/journal.pone.0255473 (PMC8330915; doi:10.1371/journal.pone.0255473)

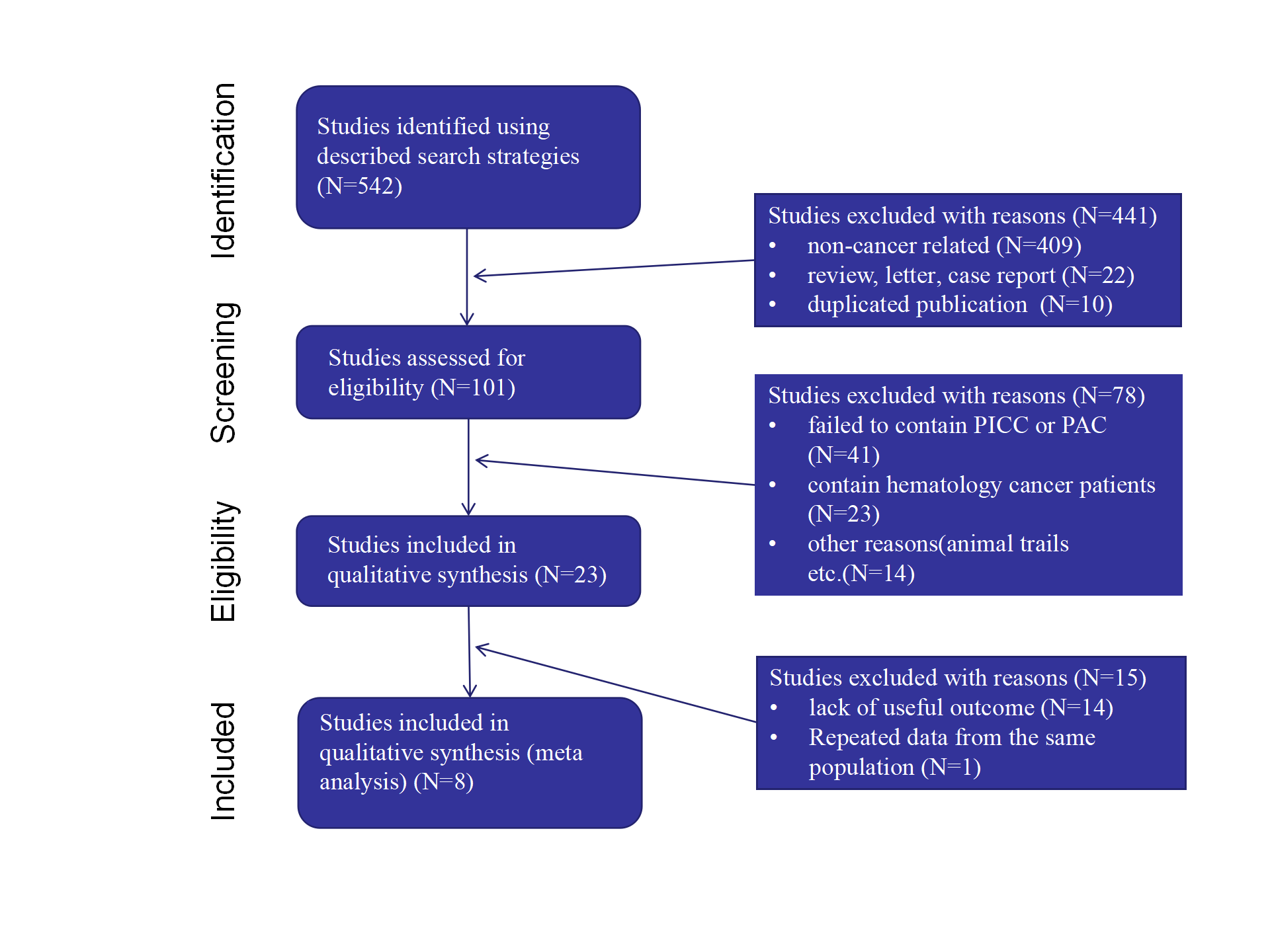

Supplement: S1 Fig — (TIF) [file pone.0255473.s002.tif]
