# Supplementary material for: Applicability of TIVAP versus PICC in non-hematological malignancies patients: A meta-analysis and systematic review
Source: PLoS One. 2021 Aug 3;16(8):e0255473. doi: 10.1371/journal.pone.0255473 (PMC8330915; doi:10.1371/journal.pone.0255473)
Supplement: S1 Table — (DOC) [file pone.0255473.s003.doc]

| Search | Query | Results |
| --- | --- | --- |
| #1 | "totally implantable vascular access device " OR "PORT" OR "Port-A-Cath" OR "PAC" OR "venous access port" OR "VAP" OR "totally implantable venous access port" OR "TIVAP" OR "TIVAD" OR "venous port access" OR "VPA" OR "central venous access device" OR"CVAD" | 99790 |
| #2 | "Catheterization, Peripheral"[Mesh] | 11886 |
| #3 | #1 AND #2 | 542 |

Table S1 Pubmed search strategies and results.
