# Supplementary material for: Applicability of TIVAP versus PICC in non-hematological malignancies patients: A meta-analysis and systematic review
Source: PLoS One. 2021 Aug 3;16(8):e0255473. doi: 10.1371/journal.pone.0255473 (PMC8330915; doi:10.1371/journal.pone.0255473)
Supplement: S1 File — (DOC) [file pone.0255473.s004.doc]

|  | Selection（4） | |  |  | Comparability(2) | | Exposure(3) |  |  |  |
| --- | --- | --- | --- | --- | --- | --- | --- | --- | --- | --- |
| Quality criteria | Case definition adequate (1) | Representativeness of the cases( 1) | Selection of controls (1) | Definition of controls (1) | Comparability based on design or analysis(2) | | Ascertainment of exposure(1) | Same method of ascertainment for cases and controls(1) | Non-Response rate(1) | Total(9) |
| Knut/2019 | ● | ● | ● | ● | ● | ○ | ● | ● | ● | 8 |
| Wang /2016 | ● | ● | ● | ● | ○ | ● | ○ | ● | ● | 7 |
| Verboom /2017 | ● | ● | ● | ● | ● | ○ | ● | ● | ○ | 7 |
| Wang/2019 | ● | ● | ● | ● | ○ | ● | ● | ● | ○ | 7 |
| Hou /2017 | ● | ● | ● | ○ | ● | ● | ● | ● | ● | 8 |
| Lefebvre/2016 | ● | ● | ● | ● | ● | ● | ● | ● | ● | 9 |
| Clemons/2020 | ● | ● | ● | ● | ● | ○ | ● | ● | ● | 8 |
| Fang /2017 | ● | ● | ● | ○ | ● | ● | ● | ● | ● | 8 |
| Lu /2017 | ● | ● | ● | ● | ● | ○ | ● | ● | ○ | 7 |
| Liu/2017 | ● | ● | ○ | ● | ● | ● | ● | ● | ● | 7 |
| Patel/2013 | ● | ● | ○ | ● | ● | ● | ● | ● | ● | 8 |
| Martella/2015 | ● | ● | ● | ● | ○ | ● | ● | ● | ● | 8 |
| Coady /2015 | ● | ● | ● | ● | ● | ○ | ● | ● | ○ | 7 |

●○
